# Intrarenal Doppler approaches in hemodynamics: A major application in critical care

**DOI:** 10.3389/fphys.2022.951307

**Published:** 2022-10-12

**Authors:** Xiaoling Qian, Junhai Zhen, Qingxiang Meng, Li Li, Jing Yan

**Affiliations:** ^1^ The Second Clinical Medical College, Zhejiang Chinese Medical University, Hangzhou, China; ^2^ Department of Critical Care Medicine, Zhejiang Hospital, Hangzhou, China; ^3^ Department of Ultrasound Medicine, Zhejiang Hospital, Hangzhou, China

**Keywords:** Doppler ultrasound, hemodynamics, renal resistive index, intrarenal venous flow pattern, renal venous stasis index

## Abstract

The treatment of severe cases usually requires multimodality hemodynamic monitoring approaches, particularly for tissue and organ perfusion tracking. Currently, only a few studies have investigated renal perfusion status at the bedside. Ultrasound has become increasingly utilized to guide the hemodynamic management of severe patients. Similarly, intrarenal Doppler (IRD) is widely used to assess renal perfusion from both the intrarenal artery and vein perspectives. The renal resistive index (RRI), which reflects the renal arterial blood flow profile, is often applied to predict the reversibility of renal dysfunction and to titrate hemodynamic support. Intrarenal venous flow (IRVF) patterns and the renal venous stasis index (RVSI), which reflects the intrarenal vein blood flow profile, are now being used to assess intravenous congestion. They may also be useful in predicting the risk of acute kidney injury and avoiding fluid overload. IRD can provide diverse and supplemental information on renal perfusion and may help to establish the early diagnosis in severe patients. This review focused on the specific operational methods, influencing factors, and applications of IRD in hemodynamics.

## 1 Introduction

The kidneys have a high blood flow perfusion rate and strong self-regulating properties; however, they are less capable of autoregulating blood flow compared with other vital organs such as the brain or heart and are more susceptible to pressure fluctuations. This characteristic renders the kidney vulnerable to damage (Liu et al., 2022). Critical illness and injury can induce hemodynamic instability, thereby causing circulation and kidney dysfunction. The resultant poor perfusion may persist for long even when systemic hemodynamics is reversed (Suarez and Busse, 2020). The final step of shock resuscitation aims to optimize single organ perfusion following the reversal of systemic hypoperfusion and restoration of microcirculatory perfusion (Noitz et al., 2020). Therefore, assessing renal perfusion is critical and should take precedence over the perfusion assessment of other organs.

There is currently no gold-standard method for determining renal perfusion in clinical practice. Although several methods for determining renal perfusion have been developed, their performance is not satisfactory. Plasma clearance of paraaminohippurate can be used to accurately measure renal blood flow; however, its performance is limited by partial renal excretion and cannot detect perfusion differences between the renal cortex and medulla. The effect of non-invasive techniques such as renal scintigraphy, computed tomography (CT), and magnetic resonance imaging (MRI) has been investigated in renal blood flow measurement. However, due to the high doses of ionizing radiation used in renal scintigraphy, the requirement for contrast agents, and the high costs or difficulty in obtaining high-quality MRI scans, these methods are not considered suitable for critically ill patients (Schneider et al., 2013).

On account of its convenience, speed, non-invasive nature, and repeatability at the bedside, ultrasound has been widely utilized to evaluate renal hemodynamics in critically ill patients (Schnell and Darmon, 2015). Conventional ultrasound is used to obtain reliable images of kidney morphology. Contrast-enhanced ultrasonography for determining renal perfusion is still in the early stage (Schneider et al., 2011). Doppler is valuable for assessing arterial or venous flow abnormalities and has been suggested for evaluating changes in intrarenal perfusion caused by diseases of the renal parenchyma and systemic hemodynamics (Di Nicolò and Granata, 2019; Pellicori et al., 2021). For intrarenal arterial Doppler, the efficacy of the renal resistive index (RRI) in predicting reversibility of renal dysfunction and titrating hemodynamic support remains controversial. For intrarenal venous Doppler, a new indicator known as the intrarenal venous flow (IRVF) pattern was developed to replace the venous impedance index (VII). Renal venous stasis index (RVSI) is another quantitative indicator to assess renal vein congestion.

In this review, we discussed the specific operational methods, influencing factors, and applications of these intrarenal Doppler technologies in hemodynamics.

## 2 Intrarenal Doppler in assessing renal arteries

**FIGURE 1:**
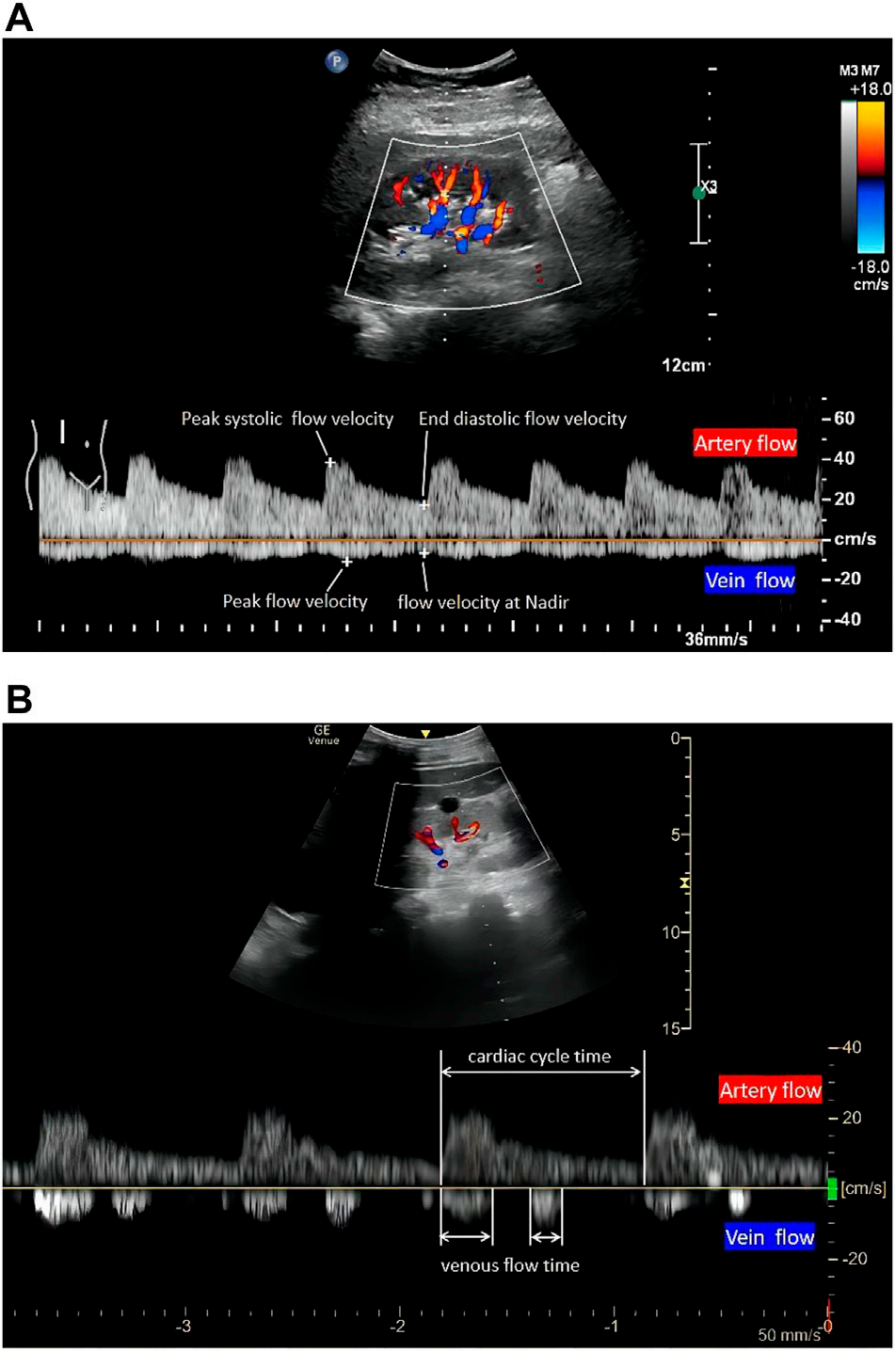
The quantitative evaluation of renal hemodynamics by pulse wave Doppler in the interlobar vessels of the right kidney. Intrarenal artery flow (upward Doppler signals) and vein flow (downward Doppler signals). A continuous venous flow pattern in **(A)** and a biphasic pattern in **(B)** (1). RRI, (peak systolic flow velocity-end diastolic flow velocity)/peak systolic flow velocity. (2) PI, (peak systolic flow velocity-end diastolic flow velocity)/mean flow velocity. (3) VII, (peak flow velocity-flow velocity at nadir)/peak flow velocity. (4) RVSI, (cardiac cycle time-venous flow time)/cardiac cycle time. RRI, renal resistive index; PI, renal pulsatility index; VII, venous impedance index; RVSI, renal venous stasis index.

Intrarenal Doppler (IRD) can be used to measure the renal artery flow velocities for the assessment of renal hemodynamics. RRI and pulsatility index (PI) are calculated by these velocities (Figure 1). Although these indices are similar, only a few studies have confirmed the value of PI in predicting long-term renal transplant function or predicting acute kidney injury (AKI) in the perioperative period (McArthur et al., 2011). As for reproducibility, PI had a much wider variation (ranging from 9.5%–22.7%) compared with RRI (ranging from 4.2%–7%) (Mastorakou et al., 1994). Thus, most authors prefer to use RRI. As the most widely used non-interventional ultrasound indicator, RRI can display the crosstalk between renal microcirculation and cardiovascular, metabolic, and inflammatory networks. Despite its limitations, it is a sensitive and reliable predictor of overall survival in patients with renal and cardiovascular disease (Di Nicolò and Granata, 2019). Hence, the periodic serial assessment may be useful to track progress and trajectory in clinic work.

### 2.1 Renal resistive index examination techniques

RRI requires a standardized study protocol, which includes five indispensable steps: first, selecting a suitable ultrasound tool is necessary. Normally, translumbar renal Doppler is the first choice for measuring RRI. However, due to the limitations associated with the patient position and operating site, transesophageal ultrasonography has also been validated to visualize the image of kidneys and measure RRI in surgery (Regolisti et al., 2017). Second, a B-mode kidney longitudinal scan needs to be visualized after detecting the suitable B-mode acoustic window with precise regulation of focus and gain. Third, it is necessary to identify interlobar arteries using colour-Doppler (Moussa et al., 2015). Fourth, pulsed wave Doppler needs to be activated. The sample volume is placed in the lumen of the vessel, and the speed-time curve is recorded through Doppler tracing. Finally, RRI is calculated using the formula (Figure 1A): RRI = (peak systolic velocity-end diastolic velocity)/peak systolic velocity. More precisely, three consecutive similar-appearing waveforms in each kidney are preferred, and the RRI value is the arithmetic average of the measurements. In addition, the right kidney is generally more accessible because the liver provides a parenchymal signal on ultrasound. If repeated measures are required, some authors have suggested limiting the inquiry to this side (Schnell and Darmon, 2012). As numerous impacting factors exist, RRI should be interpreted with caution in clinical or research settings. In adults, RRI > 0.7 is usually considered the upper normality threshold (Le Dorze et al., 2012).

### 2.2 Factors impacting renal resistive index

The definition of RRI includes the term “resistant”; however, RRI is essentially a measure of blood pulsatility. Consequently, the relationship between RRI and renal vascular resistance (VR) continues to be a source of debate. Using an *in vitro* model (comprising a pulsatile pump, blood-mimicking fluid, and varying compliance and resistance), an early study established that RRI was dependent on VR when vascular compliance was preserved but independent of VR when vascular compliance was absent (Bude and Rubin, 1999). In another study, where rabbit kidneys were perfused *ex vivo* with a pulsatile perfusion system (which can control and monitor VR, systolic and diastolic pulse pressures, and pulse kinetics), no relationship was found between RRI and VR, but a linear relationship between RRI and pulse pressure (PP) index [(systolic pressure-diastolic pressure)/systolic pressure] (Tublin et al., 1999). A clinical study involving 110 recipients of kidney transplants found that RI was associated with the pulse pressure and recipients’ age but not with the donor (Krumme et al., 1997). Thus, it can be concluded that RRI relies on vascular compliance and is mainly determined by systemic factors (PP). In their study, O'Neill (2014) also used mathematical analysis to deduce the linear relationship between RRI and pulse pressure (PP), revealing the connection between RRI and renal capillary wedge pressure (RCWP). Thus, both the intrarenal factors (RCWP) and systemic factors (PP) impact RRI.

As for systemic factors, RRI increases in direct proportion to PP, which is mainly influenced by vascular compliance and cardiac function. Specifically, RRI is inversely proportional to vascular compliance. Due to the compliance of the aortic/large arteries, the waveform of arterial blood flow during the cardiac cycle can be acquired. A decrease in systemic vascular compliance, such as arterial stiffness (physiological or pathological), may result in increased PP (elevations in systolic blood pressure and a reduction in diastolic blood pressure). Thus, age, atherosclerosis, and arterial stiffness (such as abdominal aortic calcification) lead to an increased RRI (Ohta et al., 2005; Calabia et al., 2014; Stefan et al., 2014). Second, as cardiac function affects RRI, lower heart rate and aortic insufficiency cause a greater reduction in diastolic pressure, while higher stroke volume increases systolic pressure, thus equating to an increase in PP and ultimately increasing RRI (Mostbeck et al., 1990; Kuznetsova et al., 2015; By et al., 2018).

Among intrarenal factors, RRI is primarily influenced by RCWP. Renal parenchyma injury or compression of adjacent tissues may increase RCWP, which is followed by increasing RRI; e.g., renal interstitial edema by renal parenchymal inflammation; vascular compression by the urinary obstruction; intraabdominal hypertension, and even exerting sufficient pressure on the kidney (Kirkpatrick et al., 2007; Di Nicolò and Granata, 2017; Candan et al., 2020). Systemic hemodynamic impactors such as heart failure with a higher central venous pressure (CVP) lead to systemic venous congestion, which increases the RCWP simultaneously (Mullens et al., 2009). Altogether, these factors significantly increase RCWP, which then elevates RRI. In brief, RRI reflects RCWP and, to a greater extent, systemic hemodynamic conditions, PP. The RRI should be interpreted bearing in mind the potential impact (s) of the aforementioned factors.

### 2.3 Applications of renal resistive index

RRI is a non-invasive tool that offers a new perspective on the prognosis and diagnosis of renal disease, including urinary obstruction, renal artery stenosis, diabetic nephropathy, and similar (Radermacher et al., 2001; Radermacher et al., 2002; Radermacher et al., 2003; Crutchley et al., 2009; Naesens et al., 2013). Assessing dynamic changes in RRI at the bedside RRI in critically ill patients demonstrates some benefits in predicting renal dysfunction reversibility and fluid resuscitation. In this way, RRI may be useful as a hemodynamic window for monitoring organ perfusion.

#### 2.3.1 The application of reversibility prediction in acute kidney injury

Acute kidney injury (AKI) is a common life-threatening complication of critically ill patients that has been associated with increased morbidity, mortality, and healthcare costs (Hoste et al., 2018). Early diagnosis and treatment of AKI are crucial to ensure good prognostic outcomes. Several biomarkers have been detected to predict AKI; however, it is necessary to evaluate their cost, accessibility, and a lack of real-world studies (Pickkers et al., 2021). As for the traditional markers, serum creatinine has limitations with delayed and relative features (Yao and Gao, 2021). Previous research has demonstrated that RRI is an efficient predictor of AKI reversibility (which was categorized as transient or persistent AKI) when compared to serum creatinine. In their study, Darmon et al. (2011) assessed RRI in severe patients with mechanical ventilation and discovered that when RI was >0.795, it had 92% sensitivity and 85% specificity for persistent AKI. A meta-analysis of nine studies (*n* = 176, focusing on specific populations such as patients with severe sepsis, mechanical ventilation, etc.) found that elevated RI was associated with an increased risk of persistent AKI, with a sensitivity and specificity of 0.83 when compared to serum creatinine or oliguria (Ninet et al., 2015). However, this meta-analysis had a high degree of study heterogeneity and did not take into account methodological quality. Indeed, Darmon’s subsequent study (Darmon et al., 2018) examined a larger sample size (*n* = 371) of unselected critically ill patients and found that RRI had a poor priority in predicting persistent AKI, with sensitivity and specificity of 50% (95% CI 41%–58%) and 68% (62%–74%) at the optimal cutoff (RRI = 0.71), respectively. Similar findings were subsequently reported by Renske Wiersema et al. (2020). Inconsistency in this pattern’s may be explained to some extent by the diversity of subjects, ranging from specific populations to unselected critically ill patients. Because the relative contributions of renal and hemodynamic factors can overlap, RRIs should be evaluated with caution. Combining an organ-directed marker such as RRI with a sign of shock severity (such as lactate) may be useful in the complicated condition of septic shock (Lerolle et al., 2006).

#### 2.3.2 The application of prediction and treatment in shock

During shock, the goal of resuscitation is to restore organ perfusion (Vincent and De Backer, 2013). As a result of its high blood flow perfusion and relative lack of self-regulating properties in comparison to other vital organs such as the brain or heart, the kidney is frequently injured and has a critical role in hemodynamic management during shock. During shock, an increase in PP (a decrease in diastolic pressure), impaired cardiac function, and increased RCWP (where renal parenchymal inflammation leads to renal interstitial edema) may contribute to an increase in RRI.

RRI is currently being evaluated for its predictive and therapeutic utility in these individuals with shock. Increased RRI may indicate a stage of shock in terms of early diagnosis. A study comparing RRI in 92 severe patients with and without shock patients discovered that patients with shock had a significantly higher RRI [0.751 (0.692–0.788) vs. 0.654 (0.610–0.686), *p* < 0.001] (Rozemeijer et al., 2019). Another study reported that an increased RRI (from 0.58 to 0.86) might be an earlier marker of a hemorrhagic shock than macro-circulatory parameters (Anile et al., 2019). Meanwhile, RRI is dynamic when volume status changes or vasoactive drugs apply, which may have value in guiding fluid management and titrating mean arterial pressure (MAP) in resuscitation (Deruddre et al., 2007; Schnell et al., 2013; Akaishi et al., 2020). Early studies evaluated the change in RRI following fluid challenge. While Schnell et al. (2013) revealed that RRI remained stable in patients with mechanical ventilation and fluid challenge, Moussa et al. (2015) demonstrated that RRI consistently decreased in patients with acute circulatory failure (from 0.73 ± 0.09 to 0.71 ± 0.09, *p* < 0.01). Another study revealed a correlation between RRI and the ideal MAP. Deruddre et al. (2007) assessed RRI and urine output in septic shock using an increased MAP titrated with norepinephrine, revealing that RRI may be a novel tool for determining the ideal MAP required for maintaining renal perfusion and function. Due to the multiplicity of influencing factors and the scarcity of research, the potential for RRI to provide more accurate fluid resuscitation management guidelines remains unexplored.

## 3 Intrarenal Doppler in assessing renal veins

The ability of kidneys to compensate for fluid load is affected by decreased arterial perfusion, potential intrarenal lesions and renal vein congestion. It is well known that renal congestion on the venous side is associated with a poor prognosis; meanwhile, early decongestion can improve kidney function and outcomes (Husain-Syed et al., 2021). Accordingly, it is necessary to pay more attention to evaluating renal overload or congestion. Promisingly, Doppler can also be used to determine overload or congestion. Ultrasound data from numerous organs such as the lungs, heart, inferior vena cava (IVC), internal jugular vein, and hepatic vein can be obtained very quickly to assist doctors in assessing the fluid status and making fluid management decisions (Piotrkowski et al., 2019; Elhassan et al., 2021; Koratala, 2021). Apart from the RRI obtained from intrarenal artery Doppler, intrarenal vein Doppler (IRVD) incorporates novel clinical markers, such as the venous impedance index (VII), intrarenal venous flow (IRVF), or renal venous stasis index (RVSI), which provide new insight into evaluating renal congestion or overload, diagnosing renal disease, and guiding volume management in patients with heart failure.

### 3.1 Intrarenal venous Doppler examination techniques

The first step in IRVD examination is identical to that of RRI in terms of visualizing the kidney in B mode and configuring the color Doppler features. Operators simultaneously record the interlobar arteries and veins using pulsed Doppler waveforms. The RRI is determined from the upward Doppler signal, and the venous flow determined by the downward Doppler signal is utilized to determine the VII, IRVF, or RVSI. The IRVF is a qualitative, categorical description of renal venous flow pattern; and the VII and RVSI are quantitative, calculated values based on renal venous spectral Doppler. VII is calculated using the following formula (Figure 1A): VII = (peak flow velocity-flow velocity at nadir)/peak flow velocity (Bateman and Cuganesan, 2002). VII is calculated as 1.0 in the discontinuous flow because the nadir is zero.

**FIGURE 2:**
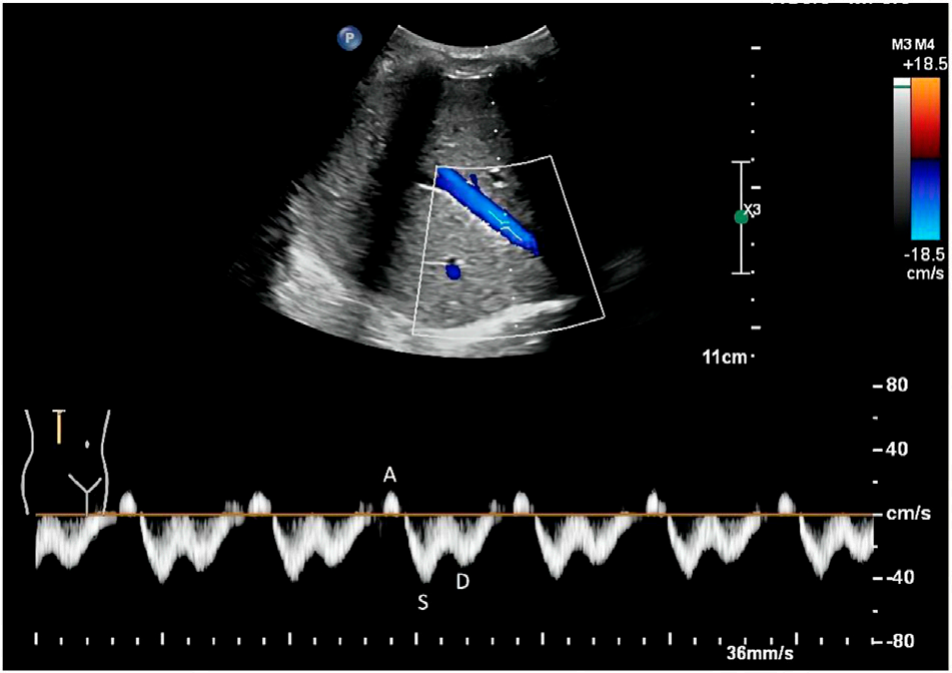
Normal hepatic vein flow pattern detected by Doppler. The hepatic vein has a triphasic waveform, which consists of an A wave above the baseline (representing atrial systole), and two waveforms below the baseline (S and D, representing venous return during ventricular systole and diastole, respectively).

**FIGURE 3:**
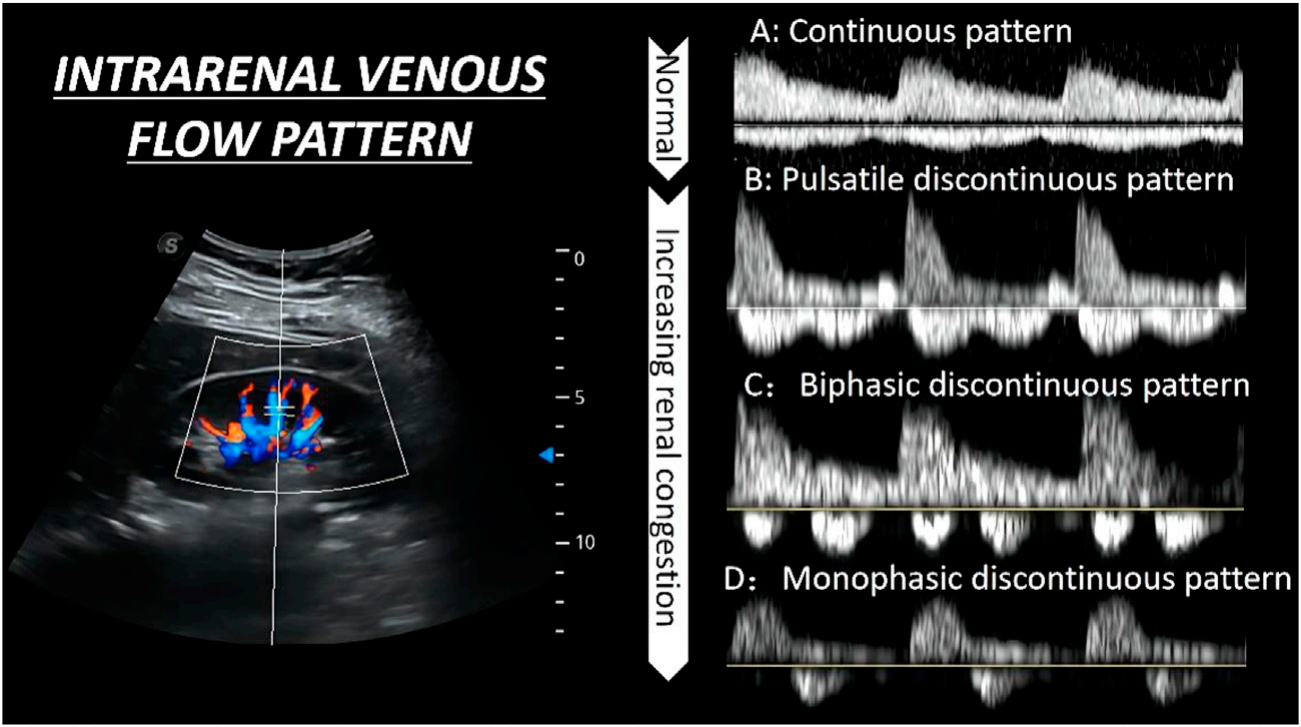
The qualitative evaluation of renal hemodynamics by pulse wave Doppler in the interlobar vessels of the right kidney. The waveform changes are divided into four flow patterns from **(A–D)**.

As for the IRVF pattern or RVSI, it is first necessary to become familiar with the formation mechanism of venous flow pattern during the cardiac cycle. Normally, the hepatic vein has a triphasic waveform produced by the change in right atrial pressure (RAP) throughout the cardiac cycle (Appleton et al., 1987) (Figure 2). This waveform consists of the baseline, an A wave above the baseline (representing atrial systole), and two waveforms below the baseline (S and D, representing venous return during ventricular systole and diastole) (Scheinfeld et al., 2009). Similar to the hepatic vein, when pulse wave Doppler imaging of the renal interlobar vascular is recorded, the artery waveform obscures waveform A, leaving only the continuous venous flow waveform with gentle undulation beneath the baseline (S and D). Currently, the IRVF pattern is regarded as a novel indicator for determining renal congestion or overload. When venous flow is unobstructed, a continuous flow pattern appears. Nevertheless, when congestion is aggravated, a discontinuous flow pattern tends to appear. The waveform changes are divided into 4 flow patterns: continuous, pulsatile discontinuous, biphasic discontinuous (with venous peaks during systole and diastole), and monophasic discontinuous (with a venous peak during diastole) (Figure 3). RVSI is a novel continuous ratio that quantifies the proportion of the cardiac cycle in which no renal venous outlet flow occurs. It is calculated using the following formula: (cardiac cycle time-venous flow time)/cardiac cycle time (Husain-Syed et al., 2019a) (Figure 1B).

### 3.2 Factors influencing intrarenal venous Doppler

Intrarenal veins act as capacitance vessels, and the vascular resistance on the venous side is normally negligible. Thus, the renal venous signal modulation is directly related to compliance, not PP (Husain-Syed et al., 2019a). Combining the formation mechanism of IRVD, the factors influencing IRVD mainly include the RAP and intrarenal venous compliance.

As RVSI is evolved from the IRVF pattern, most previous studies have focused on the IRVF pattern, which has been mainly verified depending on RAP (Scheinfeld et al., 2009; Iida et al., 2016; Husain-Syed et al., 2019a). On the one hand, high levels of RAP may decrease the speed of venous return flow during ventricular systole and comparatively increase the speed of venous flow during ventricular diastole when the tricuspid valve opens. On the other hand, increased RAP may cause renal parenchymal congestion and an increase in interstitial pressure, thus reducing intrarenal parenchymal compliance around the intrarenal vessels and increasing pressure waves or even inducing discontinuous flow pattern forward flow in the interlobar veins. In their study, Iida et al. (2016) demonstrated this phenomenon in heart failure (HF) patients where increased RAP (from 5.4 ± 2.5 mm·Hg, 9.5 ± 3.5 mm·Hg, RAP 14.9 ± 4.3 mm·Hg, *p* < 0.001) appeared in different flow pattern (from a continuous pattern, biphasic pattern to monophasic pattern), and the discontinuous IRVF pattern appeared when increased RAP level > 10 mm·Hg. Therefore other impacting factors, such as right ventricular fractional area change, moderate or severe tricuspid regurgitation, or fluid overload that was connected with the change in RAP, were associated with the monophasic pattern (Scheinfeld et al., 2009). However, the factors influencing IRVF pattern are mainly limited to the RAP, while other factors that are connected with intrarenal venous compliance (like age, atherosclerosis, and intraabdominal pressure) may also change the flow pattern. Thus, future studies focusing more on compliance are warranted.

### 3.3 Application of intrarenal venous Doppler

The first time IRVD was used to describe the hemodynamics of the intrarenal vein was using indicator VII. It was formerly described as a more accurate approach than RRI for diagnosing urinary obstruction, having a lower VII compared to unobstructed (Bateman and Cuganesan, 2002). Since then, research into VII has established a moderate correlation between VII and serum creatinine concentration in Diabetic nephropathy (Jeong et al., 2011), as well as a moderate diagnostic accuracy for AKI or preeclampsia (Bellos and Pergialiotis, 2020). However, all questions are answered about VII and kidney disease overall the relationship between hemodynamic management and the assessment of renal congestion has not been adequately investigated. In a study by Iida et al. (2016), the intrarenal hemodynamics of 224 patients with heart failure were examined to determine their prognostic implications. Results showed that 101 patients had a VII of 1.0 (96.1% in patients with a VII ≥0.53). To describe the details of discontinuous venous flow in VII of 1.0, patients were then grouped according to their IRVF pattern. A connection between RAP and the discontinuous pattern was observed. Therefore, IRVD is a suitable indicator of IRVF pattern compared with VII.

#### 3.3.1 Application of intrarenal venous flow pattern in fluid management

Right heart failure or fluid overload may cause an increase in RAP, subsequently affecting the end-organ hemodynamics such as the IVC and Hepatic Venous, resulting in a non-continuous IRVF pattern. The concept of the IRVF pattern provides a novel tool for assessing intrarenal vein congestion. In their study, Iida et al. (2016) showed that IRVF patterns were significantly correlated with clinical outcomes (a 1-year follow-up on the probability of surviving from life-threatening cardiac causes and unplanned hospitalizations because of heart failure). The monophasic IRVF pattern had a reduced prognosis compared with the other patterns (log-rank *p* < 0.001). In a different study, Nijst et al. (2017) recorded the RRI, VII, and IRVF patterns during fluid changes in healthy individuals and patients with HF. The vascular volume was expanded using 1 L of 6% hydroxyethyl starch for 3 hours. The volume was later decreased using a loop diuresis for 1 h. This study showed the IRVD indicators changed significantly in HF patients (RRI remains stable, VII has an increase after volume expanse and reverses after volume remove, IRVF was firstly more turned to discontinuous and reversed after a decrease in fluid) as compared with the indicators in healthy patients, which remained consistent in the study. In a separate study, Beaubien-Souligny et al. (2018) evaluated the relationship between IRVF and congestive heart failure, finding that severe alterations of intrarenal flow (monophasic pattern) were a marker of venous congestion in patients following cardiac surgery. Furthermore, Beaubien-Souligny et al. (2018) found that the monophasic pattern was independently associated with AKI. In summary, the IRVF pattern is strongly correlated with venous congestion after cardiac surgery. The presence of a discontinuous pattern of IRVF showed the occurrence of renal congestion as well as a poor prognosis.

In addition, it was evident that assessing intrarenal congestion may be beneficial during fluid resuscitation. Although several clinicians are aware of the dangers of fluid overload, the optimal time to discontinue resuscitation or achieve negative fluid balance remains unclear. Furthermore, assessment of the end-organ congestion may be a novel method for guiding fluid management, which includes the evaluation of renal congestion. Recently, a study conducted by Beaubien-Souligny et al. (2020) established a venous excess ultrasound (VExUS) grading system to assess venous congestion in patients undergoing cardiac surgery. VExUS grading system incorporated IVC and multiple Doppler flow patterns, including hepatic vein, portal vein, and intrarenal vein. Severe VExUS grade defines as a dilated IVC (≥2 cm) combining with at least two severe abnormalities Doppler flow patterns (the presence of a reversed systolic phase in hepatic vein Doppler, or pulsatility fraction >50% in portal vein Doppler, or only a diastolic phase in intrarenal venous Doppler). In their study, William et al. found severe VExUS grade during ICU admission, thus suggesting a high risk of postoperative AKI. The novel VExUS grading system was utilized by Rola et al. (2021) in five different clinical cases to show how it may help in the timely diagnosis of venous congestion and also provide supplementary suggestions for fluid removal. Therefore, the present study introduced a new tool for investigating the pathophysiology of cardiorenal syndromes by directly measuring intrarenal vein pressure rather than through CVP. Discontinuous IRVF, particularly the monophasic pattern and the aggravated VExUS grading system, may provide additional information to comprehensively evaluate venous congestion or fluid status and also provide guidance for timely fluid removal or discontinued fluid resuscitation.

#### 3.3.2 Application of renal venous stasis index in fluid management

Although the IRVF pattern reflects renal congestion by classifying the intrarenal venous flow, it fails to describe the continuum of renal congestion. According to Husain-Syed et al. (2019a), Doppler-derived RVSI is a continuous index for or quantifying renal congestion that increases with the severity of IRVF patterns. In their study, Husain-Syed et al. (2019b) enrolled 205 patients with pulmonary hypertension (PH) and used Cox proportional hazards models to assess the impact of quantifying IRVF, RVSI, and other factors on 1-year all-cause mortality. RVSI and RVSI independently predicted the morbidity/mortality endpoint in the third tertile, and the referent had a hazard ratio of 4.72. Receiver operating characteristic curves showed that the RVSI was a more sensitive and specific predictor of the composite endpoint compared with the individual IRVF patterns (areas under the curve: 0.789 and 0.761, respectively; *p* = 0.038). Faeq Husain-Syed also used the new index throughout the treatment procedure of a patient with right HF and major fluid overload caused by severe PH. The new index demonstrated a continuous improvement in IRVF pattern and reduction of RVSI for effective decongestion up to the normal status, from monophasic (0.74) to a continuous pattern (0). Therefore, previous studies showed that RVSI could be used as a quantitative Doppler indicator for the assessment of renal congestion and to examine the treatment response as well as guide therapies in patients with PH or HF.

## 4 Conclusion

Recently, ultrasonography has been used in ICU to assess global hemodynamics such as fluid status or volume responsiveness. There is a growing interest in assessing regional tissue perfusion, particularly in critical organs. Several methods are used to measure renal perfusion, including renal scintigraphy, CT, dynamic gadolinium contrast-enhanced MRI, cine phase-contrast MRI, and arterial spin labeling. However, these methods have limited repeatability, are costly, and are ionizing radiation. Renal Doppler is more often used to assess renal perfusion. While adopting these techniques requires well-trained operators and a learning curve, the value of RRI in predicting AKI in critically ill patients and utility in guiding resuscitation in shock have gained some recognition but remain exploratory. IRVF patterns and RVSI provide new perspectives on predicting congestion and fluid overload. As numerous contributing elements impact renal Doppler, it may be combined with other indices of renal perfusion to analyze and support clinical hemodynamic decisions comprehensively.

## Abbreviations

AKI, acute kidney injury; CD, colour Doppler; CEUS, contrast-enhanced ultrasonography; CT, computed tomography; CVP, central venous pressure; HF, hear failure; IRVD, intrarenal venous Doppler; IRD, intrarenal Doppler; IRVF, intrarenal venous flow; IVC, inferior vena cava; MAP, mean arterial pressure; MRI, magnetic resonance imaging; PH, pulmonary hypertension; PP, pulse pressure; RAP, right atrium pressure; RCWP, renal capillary wedge pressure; RRI, renal resistive index; RVSI, renal venous stasis index; SD, spectral Doppler; VExUS, venous excess ultrasound; VII, venous impedance index; VR, vascular resistance.
